# Plastrum Testudinis Extract Ameliorates Intervertebral Disc Degeneration by Suppressing NF‐κB Mediated Senescence and Inflammation

**DOI:** 10.1155/jimr/6396887

**Published:** 2026-06-25

**Authors:** Jiahui He, Yanchi Gan, Peng Zhang, Yan Gong, Zhaojun Cheng, Qi Shang, Honglin Chen, Guifeng Chen, Wenhua Zhao, Gengyang Shen, Hui Ren, Xiaobing Jiang, Henian Li

**Affiliations:** ^1^ The Second Clinical Medical College, Guangzhou Medical University, Guangzhou, Guangdong, China, gzhmc.edu.cn; ^2^ Department of Orthopedics, The Affiliated Traditional Chinese Medicine Hospital, Guangzhou Medical University, Guangzhou, Guangdong, China, gzhmc.edu.cn; ^3^ Department of Spine Surgery, The Second Affiliated Hospital of Guangzhou Medical University, Guangzhou, Guangdong, China, gzhmc.edu.cn; ^4^ Department of Orthopedics, Ruikang Hospital Affiliated to Guangxi University of Chinese Medicine, Nanning, Guangxi, China, gxtcm.com; ^5^ The Second Clinical Medical College, Guangzhou University of Chinese Medicine, Guangzhou, Guangdong, China, gzucm.edu.cn; ^6^ The First Clinical Medical College, Guangzhou University of Chinese Medicine, Guangzhou, Guangdong, China, gzucm.edu.cn; ^7^ Department of Orthopaedics, Dongguan Hospital of Traditional Chinese Medicine, Dongguan, Guangdong, China, dg-tcm.com

**Keywords:** inflammation, intervertebral disc degeneration, nucleus pulposus cells, plastrum testudinis extract, senescence

## Abstract

**Background:**

Intervertebral disc degeneration (IVDD) is a major cause of chronic low‐back pain (LBP). The inflammatory environment and senescence of nucleus pulposus cells (NPCs) within the intervertebral disc (IVD) are critical processes in IVDD. Plastrum testudinis extract (PTE) has been proven to have anti‐inflammatory qualities; however it is unknown whether it can impact the progression of IVDD and NPC senescence.

**Methods:**

We investigated the effects of PTE on NPC senescence induced by tert‐butyl hydrogen peroxide (TBHP) in vitro. Cell viability, senescence‐associated secretory phenotype (SASP), inflammatory factors, and extracellular matrix (ECM) were assessed. The NF‐κB pathway was activated using lipopolysaccharide (LPS) to explore underlying mechanisms. In vivo studies used a senile mouse with IVDD, evaluating disc structure and inflammatory factors after PTE treatment.

**Results:**

TBHP stimulation rapidly induced NPC senescence, increased inflammatory factors, and disrupted ECM anabolic balance; these effects were partially alleviated by PTE treatment. PTE inhibited NF‐κB pathway activation and prevented p65 nuclear translocation. In senile mice, PTE treatment partially preserved IVD structural integrity. Immunohistochemistry showed PTE inhibited IL‐1β expression, while immunofluorescence demonstrated increased aggrecan expression following PTE treatment.

**Conclusion:**

PTE delays IVDD progression through anti‐inflammatory effects, specifically by inhibiting NF‐κB pathway activation and reducing inflammatory factor expression. These findings suggest PTE could be a promising therapeutic agent for IVDD treatment.

## 1. Introduction

Low‐back pain (LBP) is a major medical problem worldwide, imposing a significant economic burden on society [1]. Literature reported that the prevalence of LBP is rising to 68% [2]. Intervertebral disc degeneration (IVDD) is recognized as a major cause of LBP. Of concern, degeneration of the intervertebral disc (IVD) in women begins at age 21 [3]. The prevalence of IVDD in people under 50 is over 70%, while that in people above 50 is over 90% [4]. The high prevalence of IVDD imposes a severe economic burden on patients. Hence, searching for effective IVDD prevention and treatment strategies is necessary.

The occurrence of IVDD is driven by microenvironmental changes in the IVD; however, the underlying pathophysiological mechanisms remain to be fully elucidated. Previous studies generally believe that it is a multifactorial and complicated process [5]. Various studies have shown that cellular senescence [6, 7] and inflammation [8, 9] are responsible for the degeneration of the IVD. Furthermore, loss of anabolic proteins and increased catabolism proteins after cellular senescence cause progressive damage to the IVD function and structure [10]. Thus, attenuating the inflammatory microenvironment of IVD and delaying nucleus pulposus cell (NPC) senescence are considered promising strategies for treating IVDD.

NF‐κB has been considered a seminal pathway of IVDD, and it exerts a critical role in extracellular matrix (ECM) degradation [11]. Abnormal activation of NF‐κB increases its transcriptional activity, which amplifies intercellular signals. Through multiple pathways, this leads to cell proliferation, differentiation, and apoptosis, ultimately contributing to various diseases [12]. The activated NF‐κB signaling pathway releases large amounts of inflammatory factors, such as TNF‐α and IL‐1β, to create an inflammatory microenvironment in IVD [13]. During IVDD, activation of NF‐κB directly or indirectly induces the expression of matrix‐degrading enzymes and inflammatory factors, leading to abnormal NP catabolism, NPC senescence, and ECM degradation [14, 15].

Plastrum testudinis (PT), derived from the dorsal and ventral carapace of the tortoise, *Chinemys reevesii*, is an essential traditional Chinese medicine of animal origin. PT extract (PTE) has been shown to reduce inflammation by inhibiting NF‐κB activity, which subsequently promotes cell proliferation [16]. In addition, PTE has significant antioxidant properties [17]. Given that NPC senescence and the inflammatory microenvironment are significant contributors to IVDD, we hypothesized that PTE may serve as a promising therapeutic agent for alleviating IVDD. To the best of our knowledge, few studies have investigated the association between PTE and IVDD. Therefore, in the present study, we evaluated the effects of PTE in senile mice and then investigated the molecular mechanism of PTE protection against NPC senescence.

## 2. Materials and Methods

### 2.1. Reagents and Antibodies

Fetal bovine serum (FBS) and penicillin–streptomycin were purchased from Gibco. Dulbecco’s modified Eagle’s medium/F12 (DMEM/F12) was purchased from Servicebio (Wuhan, PRC). Primary antibodies for IL‐1β, TNFα, TGFβ, p65, pp65, MMP3, MMP9, and MMP13 were purchased from Affinity Biosciences (Cincinnati, OH, USA). The antibodies for p53 and p21 were purchased from Beyotime (Shanghai, PRC). The antibodies for collagen II and aggrecan were purchased from Santa Cruz Biotechnology (Lake Placid, NY). The antibodies for β‐actin were purchased from Cell Signaling Technology (Danvers, MA, USA). The Cell Counting Kit‐8 (CCK‐8) was purchased from GLPBIO (Montclair, USA).

### 2.2. Mouse Breeding and Treatment

This research has been permitted by the Ethics Committee of our hospital (Number TCMF1‐2021026). The study was conducted according to the ARRIVE guidelines for the reporting of animal experiments. We purchased 3‐month‐old wild‐type C57BL/6 mice, which were randomly divided into three groups: young, senile, and senile + PTE. The animals were placed in a sterile circumstance with the same duration of light (12 hours/day), the same temperature (21–26°C), the same humidity (41%–70%), and adequate water and feed. After 24 months of rearing, we obtained a naturally aged mouse model of senescence, as described previously [18].

The crude drug of PT was purchased from our hospital (Batch Number KG37243537). The PT was identified as the dorsal and ventral carapace of *Chinemys reevesii* (Gray) according to the Chinese Pharmacopoeia. We prepared it based on a previous method [19]: 100 g of PT was crushed into small pieces, added to 1 L of distilled water, and boiled for 1 h. The supernatant was collected. The remaining residue was re‐extracted twice with 800 mL of distilled water, each time boiling for 1 h. The three supernatants were combined and concentrated under reduced pressure using a rotary evaporator at 60°C to obtain ~500 mL of PTE concentrate. The concentrate was lyophilized to powder and stored at −20°C until use. The yield of the lyophilized powder was ~4.5% (w/w). The PTE was standardized based on HPLC fingerprint analysis, as reported in our previous study [16], which confirmed the presence of characteristic active components and ensured batch‐to‐batch consistency. Although the full chemical profile of PTE remains to be systematically characterized by LC‐MS‐based metabolomic approaches, previous studies have identified classes of compounds in PTE, including steroids, fatty acids and their esters, amino acids, and small peptides [20, 21]. Nevertheless, as is common in traditional Chinese medicine research investigating crude extracts, the present study focuses on the holistic pharmacological effects and underlying mechanisms of PTE rather than the identification of individual active compounds. Further systematic chemical characterization—such as LC‐MS/MS analysis—will be pursued in our subsequent studies to identify the bioactive constituents responsible for the anti‐IVDD effects observed here. In this study, we treated the young and senile groups with phosphate‐buffered saline (PBS), while those in the senile + PTE group were treated with PTE. For in vitro experiments, PTE was dissolved in sterile PBS at a stock concentration of 10 mg/mL and filtered through 0.22 μm membranes. The working concentrations (0.1 and 1 μg/mL) were selected based on CCK‐8 showing no cytotoxicity. For in vivo administration, the PTE powder was freshly suspended in PBS at 400 mg/mL and administered by oral gavage at a dose of 4 g/kg/day (equivalent to 40 mg per 10 g of body weight) for 8 weeks. The gavage dose for the mouse was calculated based on the human‐to‐mouse body surface area ratio [22–24].

### 2.3. Histological Evaluation, Tissue Immunochemical Staining, and Immunofluorescence Staining

The L4/5 and L5/6 IVDs were identified based on anatomical landmarks, isolated en bloc, and fixed in 4% paraformaldehyde (pH 7.4, 24 h at 4°C). They were then decalcified in 10% EDTA (14 days with daily buffer changes) and embedded in paraffin after graded ethanol dehydration. Serial midsagittal sections (5 μm) were stained with hematoxylin–eosin (HE) to assess tissue morphology and with Safranin O/Fast Green (SO) to evaluate proteoglycan content. The degree of disc degeneration was scored by the modified histologic grading system [25]. For immunohistochemical analysis, antigen retrieval was performed in 10 mM citrate buffer (95°C, 20 min), followed by blocking with 5% BSA and incubation with anti‐IL‐1β primary antibody (1:200, Affinity Biosciences, 4°C overnight). HRP‐conjugated secondary antibody (1:500, 1 h) and DAB substrate (Beyotime) were used for detection. Immunofluorescence staining was carried out using anti‐aggrecan (1:100, Santa Cruz) with Alexa Fluor 488‐conjugated secondary antibodies (1:500, 2 h in the dark) after blocking with 10% goat serum. All stained sections were scanned using a Panoramic MIDI digital slide scanner (3DHISTECH), and quantitative analysis of the IL‐1β‐positive area (%) and aggrecan fluorescence intensity was performed using ImageJ software.

### 2.4. Cell Isolation, Culture, and Treatment

Nucleus pulposus tissues were isolated from a 3‐month‐old wild‐type C57BL/6 mouse under a microscope. The tissues were then digested with 0.25% trypsin (Gibco, USA) for 30 min and 0.1% type II collagenase (Gibco, USA) for 6 h at 37°C. After centrifugation, NPCs were harvested and incubated in a complete DMEM/F12 medium with 1% penicillin–streptomycin and 15% FBS in hypoxic circumstances containing 5% CO_2_ in a humidified incubator at 37°C. Second‐generation NPCs were collected for subsequent experiments to prevent their differentiation.

### 2.5. Cell Viability Assay

NPCs were treated with tert‐butyl hydrogen peroxide (TBHP) at various concentrations (0, 50, 100, 200, 300, and 400 μM) for 2, 4, or 6 h. Additionally, NPCs were treated with PTE at different concentrations (0, 0.01, 0.1, 1, 10, and 100 μg/mL) for 24 or 48 h. Then, CCK‐8 assays were performed to detect cell viabilities in different groups.

### 2.6. Senescence Associated β‐Galactosidase (SA‐β‐Gal) Assay

Mouse NPCs were inoculated into six‐well plates. After stimulation with TBHP in the presence or absence of PTE at the indicated concentrations, mouse NPCs were washed three times with PBS. The cells were then fixed with 1 mL fixative solution (Beyotime) for 15 min at room temperature, stained with 1 mL β‐galactosidase staining solution (Beyotime), and incubated overnight at 37°C. Then, we acquired and analyzed images under a Leica microscope.

### 2.7. Western Blotting Analysis

Following treatment with TBHP in the presence or absence of PTE at indicated concentrations, mouse NPCs (2 × 10^6^ cells) were seeded into 100 mm culture dishes. Proteins were then extracted by adding 200 μL of RIPA lysis buffer supplemented with phosphatase and protease inhibitors (Beyotime) per dish. Protein bands were transferred onto polyvinylidene fluoride membranes (Bedford, MA, USA) via electrophoresis and wet transfer. The membranes were then blocked with QuickBlock Western blocking solution (Beyotime) for 60 min at room temperature. Primary antibodies were added and incubated overnight at 4°C in a shaker. Then, the corresponding secondary antibody was added and incubated in a shaker at 26°C for 1.5 h. A gel imaging system subsequently detected the antibody reactivity level (Bio‐Rad Laboratories, Hercules, CA, USA). Finally, the grayscale values were quantitated using ImageJ software.

### 2.8. Network Pharmacology Analysis

#### 2.8.1. Retrieval of PTE‐Associated Components and Targets

PTE‐associated bioactive components and targets were retrieved from the BATMAN‐TCM database (http://bionet.ncpsb.org/batman-tcm/) with a score cutoff of 10 and with “popular organisms” restricted to humans [26]. Notably, oral bioavailability (OB) is the principal direction in drug studies. OB ≥ 30% is recommended as a selection criterion for active compounds within PTE.

#### 2.8.2. Retrieval of NPC Senescence‐Related Genes

The key word: “nucleus pulposus cell senescence” was searched in the human genetic database GeneCards (https://www.genecards.org/) [27] with the species set as ‘*Homo sapiens*’ and a minimum relevance score of 3.

#### 2.8.3. Overlapped Target Proteins (OTPs) and Protein–Protein Interaction (PPI) Analysis of OTPs

We used R software (v3.6.1) to identify the intersection between PTE‐associated and NPC‐associated targets, yielding the OTPs. To explore the connections between the overlapping target genes, the STRING database (https://string-db.org/) was employed to retrieve PPI data, restricting the species to *Homo sapiens*. A PPI network involving the intersecting target proteins was then built using Cytoscape software (Version 3.7.2; http://www.cytoscape.org/). Network topology analysis was performed with CytoHubba (a Cytoscape plugin), which enabled the calculation of the degree for each node, thereby identifying the core hub targets.

#### 2.8.4. Gene Ontology (GO) Enrichment Analysis and Kyoto Encyclopedia of Genes and Genomes (KEGG) Pathway Analysis

We performed GO enrichment analysis concerning biological process (BP) via the clusterProfiler package (R3.6.1) and selected the 20 representative enrichment results with *p* < 0.05. Next, we carried out KEGG analysis of OTPs using the clusterProfiler package (R3.6.1) and extracted the top 30 items of significant enrichment results (*p* < 0.05).

### 2.9. Cell Immunofluorescence Staining

NPCs were cultured in 24‐well plates (4 × 10^4^ cells/well) and then fixed with 4% paraformaldehyde for 15–20 min. After washing with PBS containing 0.1% Tween‐20 (PBST), the samples were incubated with 0.3% Triton X‐100 for 15 min and then blocked with QuickBlock Blocking Buffer for Immunol Staining (Beyotime) for 60 min. The cells were treated with primary antibodies against p65 (1:100) and pp65 (1:100) overnight at 4°C. Secondary antibody was then incubated at 26°C for 1 h. Fluorescence images were obtained by fluorescence microscopy (Leica).

### 2.10. Statistical Analysis

All statistical analyses were performed using SPSS 26.0 (SPSS Institute, Chicago, IL, USA). Data were presented as mean ± standard deviation. For comparisons among multiple groups, one‐way ANOVA followed by Dunnett’s or Tukey’s post hoc test was employed, as indicated in the figure legends. Statistical significance was set at *p*  < 0.05.

## 3. Results

### 3.1. TBHP Induces NPC Senescence

**Figure 1:**
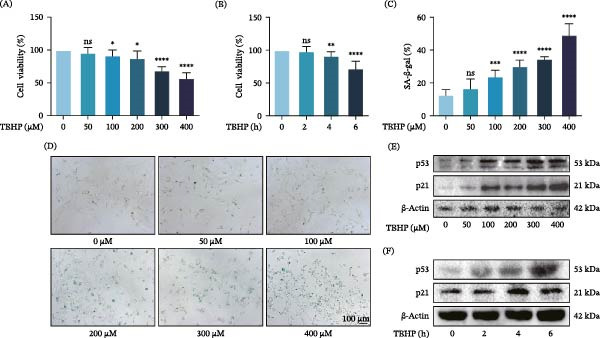
TBHP induces NPC senescence. (A, B) CCK‐8 results show cell viability of mouse NPCs after stimulation with different concentrations of TBHP for 4 h and intervention with 100 μM for different duration (*n* = 6 technical replicates per experiment). (C, D) SA‐β‐gal staining of NPCs stimulated with different concentrations of TBHP for 4 h and quantitative analysis of SA‐β‐gal‐positive cells (*n* = 4 technical replicates per experiment). (E, F) Western blot analysis of p53 and p21 expression in NPCs after TBHP treatment at indicated concentrations for 4 h (E) or with 100 μM TBHP for the indicated durations (*n* = 4 biological replicates). All data are presented as mean ± SD. Statistical significance was analyzed by one‐way ANOVA followed by Dunnett’s post hoc test comparing each group to the control group (0 μM or 0 h).  ^∗^ *p*  < 0.05,  ^∗∗^ *p*  < 0.01,  ^∗∗∗^ *p*  < 0.001,  ^∗∗∗∗^ *p*  < 0.0001.

To construct an NPC senescence model, we used TBHP to intervene in mouse‐derived NPC [28]. The CCK‐8 assay revealed that cell viability began to decrease following 4 h of TBHP treatment at a concentration of 100 μM. Cell viability was correlated with the concentration and time of TBHP stimulation. A higher concentration and longer exposure to TBHP resulted in a more pronounced reduction in NPC viability. (Figure 1A,B). Hence, we chose a 100 μM TBHP intervention for 4 h as the subsequent experimental condition.

To confirm that TBHP induces NPC senescence, we examined the expression of SA‐β‐gal and senescence‐associated proteins in NPC after TBHP intervention. The SA‐β‐gal assay showed that increasing TBHP concentrations led to a higher percentage of β‐galactosidase‐positive cells (Figure 1C,D). In addition, Western blot showed that the expression levels of senescence‐related proteins p21 and p53 elevated with increasing TBHP concentration or longer stimulation time (Figure 1E,F).

### 3.2. PTE Reverses TBHP‐Induced NPC Senescence

**Figure 2:**
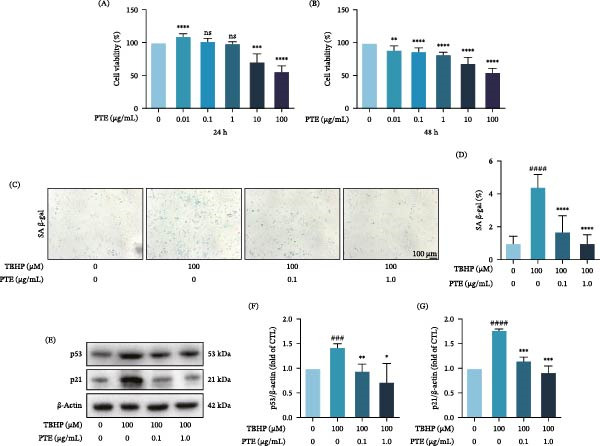
PTE reverses TBHP‐induced senescence in NPC. (A, B) CCK‐8 results show the cell viability of NPC after treatment with different concentrations of PTE for 24 and 48 h. Differences between 0 μg/mL and other concentrations were compared (*n* = 6 technical replicates per experiment). Statistical significance was determined by one‐way ANOVA with Dunnett’s post hoc test, comparing each PTE‐treated group to the control group (0 μg/mL): ns, not significant,  ^∗^ *p*  < 0.05,  ^∗∗^ *p*  < 0.01,  ^∗∗∗^ *p*  < 0.001,  ^∗∗∗∗^ *p*  < 0.0001. (C, D) SA‐β‐gal staining of NPCs treated with PTE and TBHP and quantitative analysis of positive cells (*n* = 4 technical replicates per experiment). (E–G) The protein content of p53 and p21 in NPC treated with TBHP and different concentrations of PTE (*n* = 3 biological replicates). These values were presented as mean ± SD. Statistical significance was determined by one‑way ANOVA followed by Dunnett’s post hoc test: # indicates comparison between the control (CTL) group and the TBHP group;  ^∗^ indicates comparison between the TBHP group and the TBHP + PTE group. ns, not significant,  ^∗^/# *p*  < 0.05,  ^∗∗^/## *p*  < 0.01,  ^∗∗∗^/### *p*  < 0.001,  ^∗∗∗∗^/#### *p*  < 0.0001.

PTE has antioxidant and anti‐inflammatory effects [16, 17]. We determined the cytotoxicity of PTE by the CCK‐8 assay to clarify its effect on NPC cell viability. Treatment with 0.1 or 1 μg/mL PTE for 24 h had no significant effect on NPC viability. All concentrations had an impact on cell viability after PTE intervention for 48 h (Figure 2A,B). The expression of SA‐β‐gal and senescence‐associated proteins p21 and p53 was detected after 4 h of 100 μM TBHP stimulation and 24 h of treatment with 0.1 or 1 μg/mL PTE. The results showed that the number of SA‐‐gal‐β‐gal‐positive cells increased after TBHP intervention, while PTE treatment could relatively reduce the number of positive cells (Figure 2C,D). The expression of senescence‐related proteins p21 and p53 was increased in NPC after TBHP intervention, and PTE treatment partially reduced the expression of senescence‐related proteins (Figure 2E–G).

### 3.3. The Anabolic and Catabolic Balance of ECM was Disrupted When NPC Senescence Occurs, and PTE Ameliorates This Change

**Figure 3:**
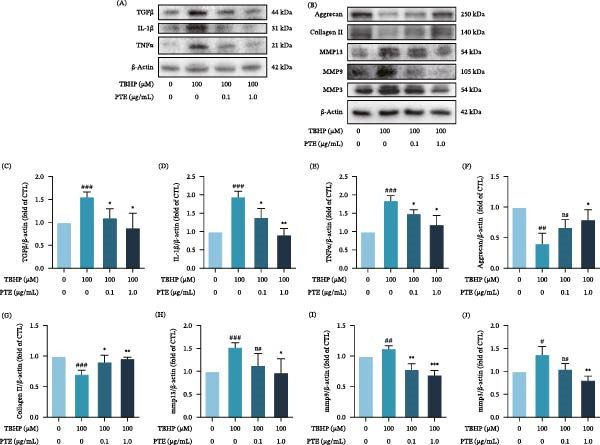
PTE inhibits inflammation in NPC and maintains ECM homeostasis. NPC was incubated with 0.1 and 1.0 μg/mL PTE for 24 h and 100 μM TBHP for 4 h. (A, C–E) After the above treatment, the protein content of inflammatory factors TGFβ, IL‐1β, and TNFα in NPC (*n* = 3 biological replicates). (B, F–J) After the above treatment, the protein content of ECM anabolic and catabolic proteins in NPC (*n* = 3 biological replicates). These values were presented as mean ± SD. Statistical significance was determined by one‑way ANOVA followed by Dunnett’s post hoc test: # indicates comparison between the control (CTL) group and the TBHP group;  ^∗^ indicates comparison between the TBHP group and the TBHP + PTE group. ns, not significant,  ^∗^/# *p*  < 0.05,  ^∗∗^/## *p*  < 0.01,  ^∗∗∗^/### *p*  < 0.001,  ^∗∗∗∗^/#### *p*  < 0.0001.

Senescence‐associated secretory phenotype (SASP) refers to cytokines secreted by senescent cells. It is closely associated with various physiological processes and aging‐related diseases [29, 30]. Following TBHP stimulation of NPCs, we observed increased secretion of SASP factors such as TGF‐β, IL‐1β, and TNFα, whereas PTE treatment effectively reduced their expression levels (Figure 3A,C–E). The ECM metabolic imbalance induced by TBHP, characterized by upregulated catabolic enzymes (MMP13, MMP9, and MMP3) and downregulated anabolic components (aggrecan and collagen II), was substantially improved by PTE intervention. Notably, PTE demonstrated a dual regulatory effect, simultaneously suppressing the overexpression of matrix‐degrading enzymes while restoring the production of key ECM structural proteins, thereby reestablishing ECM homeostasis in senescent NPCs (Figure 3B,F–J).

### 3.4. Network Pharmacology Reveals That NF‐κB Pathway was Associated With PTE Reversal of NPC Senescence Process

Using network pharmacology, we analyzed PTE‐related components and target proteins. From the BATMAN‐TCM database, we obtained 6 bioactive components (threonine, aspartic acid, calcium carbonate, methionine, leucine, and phenylalanine) and 342 PTE targets. We obtained a total of 317 NPC‐related target proteins after analyzing the target information of NPC. We took the intersection between PTE‐ and NPC‐associated targets as OTPs comprised of 11 targets (Table 1 and Figure 4A). The PPI network of OTPs is plotted in Figure 4B. The top five targets in terms of degree are shown in Figure 4C. GO enrichment analysis yielded a total of 734 BP entries. Notably, 20 entries were screened, mainly correlated with oxidative stress, inflammation regulation, regulation of NF‐κB transcription factor activity, and NF‐κB signaling, which were closely associated with the pathological process of NPC (Figure 4D). We obtained a total of 67 significant pathways by KEGG pathway analysis, and we present 30 of them with top *p* values (Figure 4E). The results showed that the NF‐κB signaling pathway might play an essential role in the effect of PTE on NPC.

Figure 4PTE suppresses the NF‐κB signaling pathway. (A) Venn diagram of PTE‐NPC intersection targets. (B) The PPI network of OTPs. (C) The top five targets in terms of degree. (D) GO.BP enrichment analysis. (E) KEGG Pathway Analysis. (F–H) Results of p65 and phosphorylated p65 protein content and immunofluorescence in NPC after incubation with 0.1 and 1.0 μg/mL PTE for 24 h and 100 μM TBHP for 4 h (*n* = 3 biological replicates). Statistical significance was determined by one‑way ANOVA followed by Dunnett’s post hoc test. Differences between the CTL group and the TBHP group were presented as #. Differences between TBHP group and TBHP + PTE group were presented as  ^∗^. (I–K) p65 and phosphorylated p65 protein content and immunofluorescence after stimulation with the NF‐κB activator lipopolysaccharide (LPS) and incubation with PTE (*n* = 3 biological replicates). Statistical significance was determined by one‑way ANOVA followed by Dunnett’s post hoc test. Differences between the CTL group and LPS group were presented as #. Differences between the PTE group and the LPS + PTE group were presented as  ^∗^. ns, not significant,  ^∗^/# *p*  < 0.05,  ^∗∗^/## *p*  < 0.01,  ^∗∗∗^/### *p*  < 0.001,  ^∗∗∗∗^/#### *p*  < 0.0001.

**Figure:**
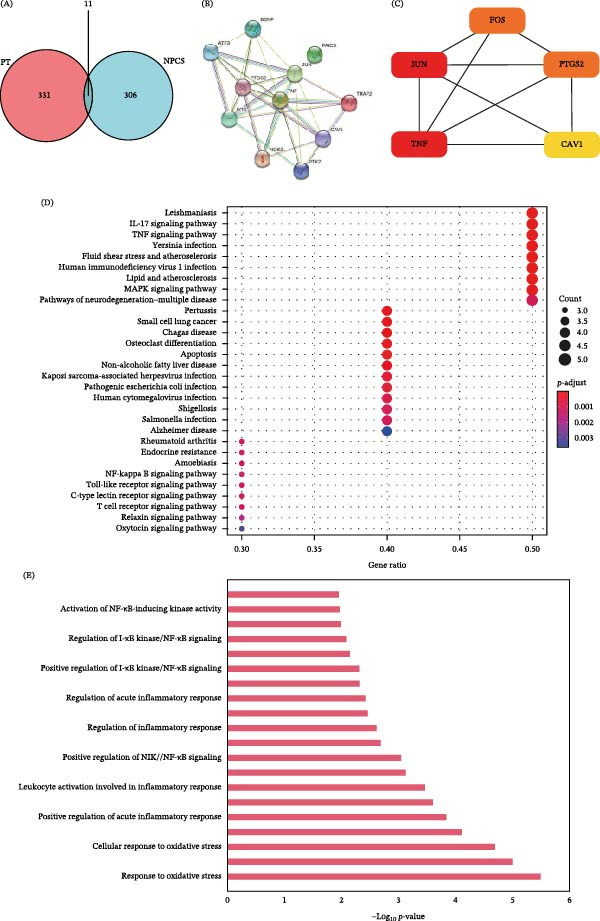

**Figure:**
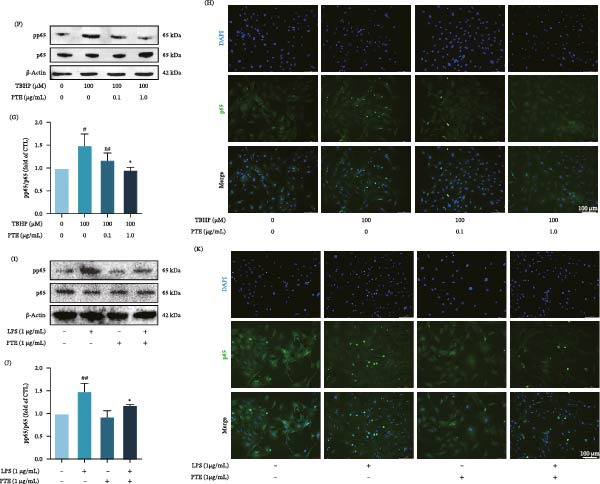

**Table 1:** Potential target genes of PTE against NPC.

Number	Gene
1	PAICS
2	PTGS2
3	TRAF2
4	NOS2
5	CAV1
6	PTK2
7	ATF3
8	TNF
9	JUN
10	FOS
11	BDNF

### 3.5. PTE Inhibits the Activation of NF‐κB Pathway and Ameliorates TBHP‐Induced NPC Senescence

Through network pharmacology, we found that the therapeutic effect of PTE on TBHP‐induced NPC senescence may work via the NF‐κB pathway. Therefore, during this process, we examined NF‐κB p65 and its phosphorylated protein levels. Western blot results showed that after TBHP stimulation, NF‐κB p65 phosphorylation levels increased in NPC, while they were significantly diminished after PTE treatment (Figure 4F,G). In addition, immunofluorescence of NPC revealed that TBHP stimulates NF‐κB p65 nuclear translocation, which can be partially inhibited by PTE (Figure 4H). Subsequently, we performed rescue experiments. Lipopolysaccharide (LPS), an agonist of NF‐κB p65, at a concentration of 1 μg/mL was added to activate the NF‐κB p65 pathway, and we found that the NF‐κB p65 phosphorylation level was increased and the NF‐κB p65 pathway was successfully activated. In addition, NPC was treated with PTE and LPS, and the results showed that the expression of phosphorylated NF‐κB p65 was reduced and the NF‐κB p65 pathway was successfully inhibited (Figure 4I,J). The same results were obtained from immunofluorescence experiments, whereby NF‐κB p65 translocation into the nucleus was increased after LPS stimulation, and it was significantly inhibited after PTE treatment (Figure 4K). Consequently, PTE delays TBHP‐induced NPC senescence by partially inhibiting the NF‐κB p65 pathway.

### 3.6. PTE Inhibits IVDD in Senile Mouse In Vivo

**Figure 5:**
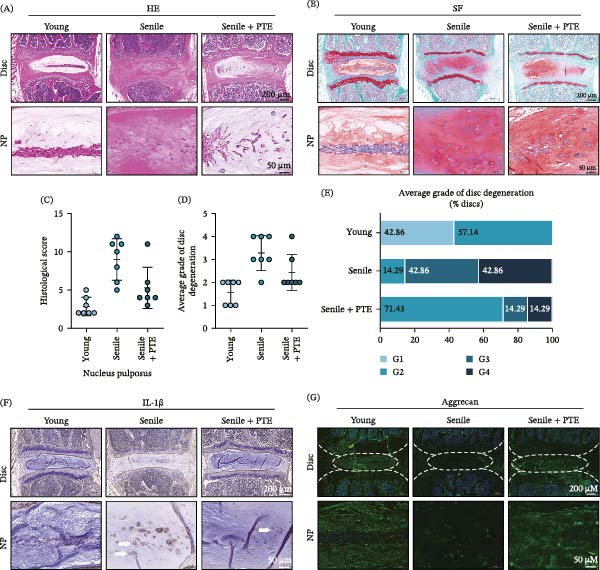
PTE treatment alleviates IVDD in mice in vivo. (A, B) Representative HE and SO staining of intervertebral disc samples from mice in the young group, senile group, and senile + PTE group. Each staining experiment was repeated three times. (C) Histological score of nucleus pulposus of three groups (7 animals per group). (D, E) Average grade of disc degeneration (7 animals per group). (F) Immunohistochemical staining of IL‐1β in intervertebral disc samples. Each staining experiment was repeated three times. (G) Immunofluorescence of aggrecan in intervertebral disc samples. Each staining experiment was repeated three times.

After 24 months of raising, we obtained senile mice. Senile mice show severe degeneration of IVDs in vivo compared to young mice. HE staining demonstrated that the overall structure of the IVD in the young mouse was well‐defined with clear boundaries between tissues. The NP tissue has a large number of NPCs in a stellar shape. Annulus fibrosus (AF) tissues were well organized, with no obvious rupture. The boundary between the NP and AF was clear. The IVD structure was disordered, and intertissue boundaries were unclear in the senile mouse. The number of cells in the NP tissue was significantly diminished, with disorganized arrangement, apparent tissue scarring, increased fibrous tissue, and unrecognizable demarcation with the AF tissue. The layers of fibers in the AF were indistinguishable. In contrast, the disc structure of the senile mouse in the PTE‐treated group was slightly disorganized, and tissue boundaries were discernible. The proteoglycan matrix around the NP tissue was slightly disorganized, cells were scattered in the middle of the NP tissue, and cell morphology did not differ significantly from normal mice. The boundaries between fibers in each layer of the AF tissue and between the AF and NP tissues were clear (Figure 5A). SO staining revealed that proteoglycan was lost in NP tissues of senile mice, while PTE‐treated senile mice still had comparatively more proteoglycan in the IVD (Figure 5B). According to the modified histological grading system, only mild degenerative changes were observed in the NP tissue of young mice. In contrast, the histological grade of NP degeneration was significantly higher in the senile mouse. This increase was markedly attenuated in mice treated with PTE (Figure 5C). Regarding the Pfirrmann grading for IVDD, the young mouse exhibited only Grades 1 and 2 degeneration. In contrast, the majority of senile mice reached Grades 3 and 4, with only a minority at Grade 2. Following PTE treatment, the degenerative grade in most mice reverted to Grade 2, with only a few cases remaining at Grades 3 and 4 (Figure 5D,E).

### 3.7. PTE Inhibits Inflammatory Factor and Regulates ECM Homeostasis in Senile Mouse IVD

Immunohistochemical staining of IVD tissue sections revealed no significant IL‐1β expression in the IVD of the young mouse. In contrast, IL‐1β expression was significantly increased in the disc tissues of senile mice and decreased in PTE‐treated mice (Figure 5F). Aggrecan was abundantly present in the IVDs of the young mouse, whereas it was expressed at limited levels in the senile mouse. PTE promoted aggrecan expression in the IVDs of senile mice (Figure 5G).

## 4. Discussion

IVDD is an aging‐related disease characterized by an imbalance between anabolism and catabolism within the disc, driving NPCs into senescence [31–34]. Oxidative stress and inflammation are key triggers of NPC senescence [35–37]. In this study, TBHP effectively induced NPC senescence in mouse cells, as evidenced by increased SA‐β‐gal activity and upregulated p53/p21 expression, consistent with previous findings in rat and human NPCs [38–40].

Inflammation not only triggers but also exacerbates IVDD [41, 42]. Senescent NPCs acquire a SASP, releasing pro‐inflammatory cytokines such as TNFα, IL‐1β, and TGFβ, which further accelerate disc degeneration [43–45]. Our results showed that PTE significantly reduced SASP upregulation, decreased p21/p53 expression, lowered SA‐β‐gal positivity, and restored the balance of ECM by increasing aggrecan and collagen II while suppressing MMP3/9/13 [37, 45]. These findings indicate that PTE delays NPC senescence primarily through anti‐inflammatory effects.

PT has been used in traditional Chinese medicine for thousands of years to treat LBP. Our previous studies demonstrated that PTE possesses anti‐inflammatory and antioxidant properties [16, 17], and similar nucleus pulposus–protective effects have been reported for other herbal constituents such as morroniside [46]. Notably, PTE has been reported to improve antioxidant status in NPCs [47], inhibit inflammatory signaling [16], and even ameliorate age‐related Parkinson’s disease [48]. Together with our in vivo results showing that PTE partially preserved the disc structure, reduced IL‐1β expression, and restored aggrecan in aged mice, these data support the therapeutic potential of PTE for IVDD. Mechanistically, network pharmacology and experimental validation revealed that PTE suppresses the NF‐κB pathway. PTE inhibited TBHP‐induced nuclear translocation of the p65 subunit, thereby reducing inflammatory factor transcription. This mechanism underlies the anti‐inflammatory and antisenescence effects of PTE [11, 49, 50].

Despite these promising findings, several challenges remain before clinical translation. The avascular nature of the IVD limits drug delivery [33], and the pharmacokinetic properties of PTE—particularly its bioavailability and ability to penetrate disc tissue—require further investigation. Future studies should compare systemic versus local administration to determine the optimal route.

Regarding study limitations, the network pharmacology analysis identified only 11 overlapping targets between PTE and NPC senescence due to stringent screening criteria (BATMAN‐TCM score ≥ 10) and a highly specific keyword. While this rigorous approach enhances confidence in these high‐confidence targets (e.g., TNF, JUN, FOS, PTGS2, and CAV1), it may overlook broader interactions. Future studies should employ complementary databases and relaxed thresholds to capture a wider range of potential targets. Additionally, our in vivo model used naturally aged mice, which mimics human aging but does not account for other IVDD etiologies such as mechanical injury or genetic predisposition. Validation in additional models is warranted.

## 5. Conclusion

**Figure 6:**
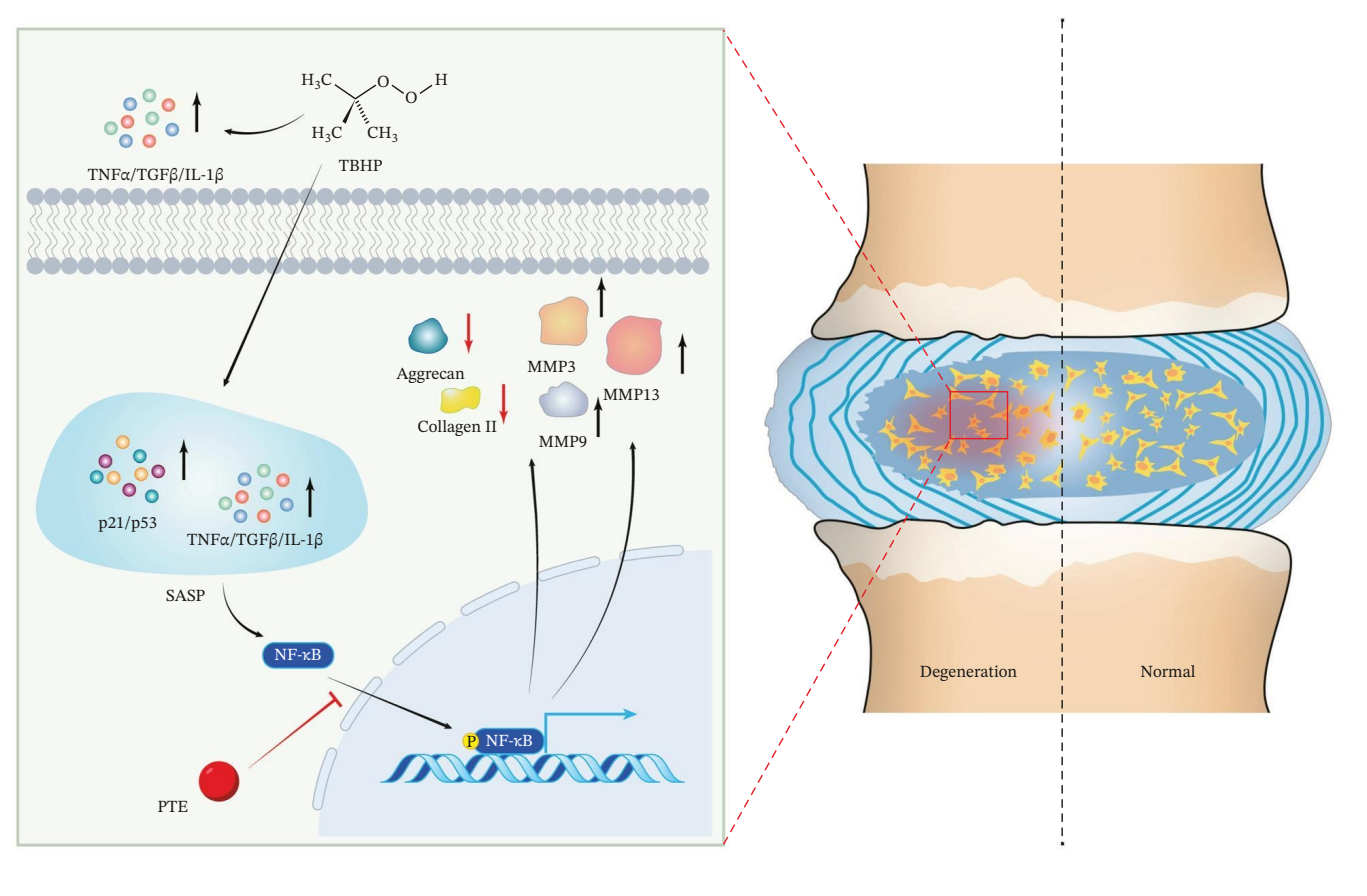
Schematic chart.

Our findings demonstrate that PTE exerts protective effects against IVDD through multiple mechanisms. The current study reveals that (1) TBHP‐induced NPC senescence is effectively mitigated by PTE treatment; (2) PTE attenuates disc inflammation by inhibiting NF‐κB p65 nuclear translocation and reducing SASP secretion; and (3) PTE promotes ECM homeostasis by enhancing collagen II and aggrecan production (Figure 6).

While these results highlight PTE’s potential as a therapeutic candidate for IVDD, further studies are needed to determine the optimal delivery method (systemic vs. local administration) for clinical translation. Particularly, the pharmacokinetic properties of PTE and its ability to penetrate the avascular disc microenvironment require careful evaluation in future preclinical and clinical investigations. These findings provide a foundation for developing PTE‐based strategies to slow or prevent disc degeneration progression.

**Nomenclature**
IVDD: Intervertebral disc degeneration
IVD: Intervertebral disc
NPCs: Nucleus pulposus cells
PT: Plastrum testudinis
PTE: Plastrum testudinis extract
TBHP: Tert‐butyl hydroperoxide
NF‐κB: Nuclear factor κB
ECM: Extracellular matrix
LPS: Lipopolysaccharide
SASP: Senescence‐associated secretory phenotype
SA‐β‐gal: Senescence‐associated β‐galactosidase
HE: Hematoxylin–eosin
SO: Safranin O/fast green
DMEM/F12: Dulbecco’s modified eagle’s medium/nutrient mixture F‐12
FBS: Fetal bovine serum
CCK‐8: Cell counting kit‐8
RIPA: Radioimmunoprecipitation assay
GO: Gene ontology
KEGG: Kyoto encyclopedia of genes and genomes
BP: Biological process
OTPs: Overlapped target proteins
LBP: Low back pain
AF: Annulus fibrosus
NP: Nucleus pulposus
IL‐1β: Interleukin‐1 β
TNFα: Tumor necrosis factor α
TGFβ: Transforming growth factor β
MMP: Matrix metalloproteinase
p65: NF‐κB p65 subunit
pp65: Phosphorylated NF‐κB p65 subunit
p53: Tumor protein p53
p21: Cyclin‐dependent kinase inhibitor 1A.

## Author Contributions

All authors contributed to the study conception and design. Material preparation, data collection, and analysis were performed by Jiahui He, Yanchi Gan, Peng Zhang, Yan Gong, Hui Ren, Gengyang Shen, Henian Li, Xiaobing Jiang, Zhaojun Cheng, Honglin Chen, Guifeng Chen, Qi Shang, and Wenhua Zhao. Validation was conducted by Jiahui He, Yanchi Gan, Zhaojun Cheng, Honglin Chen, Guifeng Chen, Qi Shang, and Wenhua Zhao. Formal analysis was performed by Yan Gong. The first draft of the manuscript was written by Jiahui He, and all other authors commented on the previous versions. Supervision and funding acquisition were led by Xiaobing Jiang, Hui Ren, Gengyang Shen, Henian Li, and Jiahui He.

## Funding

This work was supported by the National Natural Science Foundation of China (Grants 82274542, 82274615, and 82205137), the Dongguan Science and Technology of Social Development Program (Grant 20221800906042), the Young Science and Technology Talents Fund of The Affiliated TCM Hospital of Guangzhou Medical University (Grant 2023RC10), and the 2024 Doctoral Student Innovation Capacity Enhancement Project of “Open Bidding for Selecting the Best Candidates” from the Guangzhou University of Chinese Medicine (Grant A3‐0317‐24‐429‐006).

## Disclosure

All authors read and approved the final manuscript.

## Ethics Statement

This research has been permitted by the Ethics Committee of our institution (Number TCMF1‐2021026).

## Conflicts of Interest

The authors declare no conflicts of interest.

## Data Availability

The datasets generated and analyzed during the current study are not publicly available due to institutional data policies but are available from the corresponding author upon reasonable request.

## References

[1] Kahere M. , Ngcamphalala C. , Östensson E. , Ginindza T. , and Huang K.-C. , The Economic Burden of Low Back Pain in KwaZulu-Natal, South Africa: A Prevalence-Based Cost-of-Illness Analysis From the Healthcare Provider’s Perspective, PLoS ONE. (2022) 17, no. 10, 10.1371/journal.pone.0263204, e0263204. 36227919 PMC9560048

[2] Wong C. K. , Mak R. Y. , and Kwok T. S. , et al.Prevalence, Incidence, and Factors Associated With Non-Specific Chronic Low Back Pain in Community-Dwelling Older Adults Aged 60 Years and Older: A Systematic Review and Meta-Analysis, The Journal of Pain. (2022) 23, no. 4, 509–534, 10.1016/j.jpain.2021.07.012. 34450274

[3] Näther P. , Kersten J. F. , Kaden I. , Irga K. , and Nienhaus A. , Distribution Patterns of Degeneration of the Lumbar Spine in a Cohort of 200 Patients With an Indication for Lumbar MRI, International Journal of Environmental Research and Public Health. (2022) 19, no. 6, 10.3390/ijerph19063721, 3721. 35329406 PMC8951543

[4] Oichi T. , Taniguchi Y. , Oshima Y. , Tanaka S. , and Saito T. , Pathomechanism of Intervertebral Disc Degeneration, JOR SPINE. (2020) 3, no. 1, 10.1002/jsp2.1076, e1076. 32211588 PMC7084053

[5] Binch A. L. A. , Fitzgerald J. C. , Growney E. A. , and Barry F. , Cell-Based Strategies for IVD Repair: Clinical Progress and Translational Obstacles, Nature Reviews Rheumatology. (2021) 17, no. 3, 158–175, 10.1038/s41584-020-00568-w. 33526926

[6] Lim S. , An S. B. , and Jung M. , et al.Local Delivery of Senolytic Drug Inhibits Intervertebral Disc Degeneration and Restores Intervertebral Disc Structure, Advanced Healthcare Materials. (2022) 11, no. 2, 10.1002/adhm.202101483, 2101483. 34699690

[7] Yang S. , Zhang F. , Ma J. , and Ding W. , Intervertebral Disc Ageing and Degeneration: The Antiapoptotic Effect of Oestrogen, Ageing Research Reviews. (2020) 57, 10.1016/j.arr.2019.100978, 100978. 31669486

[8] Chen S. , Lei L. , and Li Z. , et al.Grem1 Accelerates Nucleus Pulposus Cell Apoptosis and Intervertebral Disc Degeneration by Inhibiting TGF-β-Mediated Smad2/3 Phosphorylation, Experimental & Molecular Medicine. (2022) 54, no. 4, 518–530, 10.1038/s12276-022-00753-9. 35440754 PMC9076866

[9] DiStefano T. J. , Vaso K. , Danias G. , Chionuma H. N. , Weiser J. R. , and Iatridis J. C. , Extracellular Vesicles as an Emerging Treatment Option for Intervertebral Disc Degeneration: Therapeutic Potential, Translational Pathways, and Regulatory Considerations, Advanced Healthcare Materials. (2022) 11, no. 5, 10.1002/adhm.202100596, e2100596. 34297485 PMC8783929

[10] Guerrero J. , Häckel S. , and Croft A. S. , et al.The Nucleus Pulposus Microenvironment in the Intervertebral Disc: The Fountain of Youth?, European Cells and Materials. (2021) 41, 707–738, 10.22203/eCM.v041a46. 34128534

[11] Zhang G.-Z. , Liu M.-Q. , and Chen H.-W. , et al.NF-κB Signalling Pathways in Nucleus Pulposus Cell Function and Intervertebral Disc Degeneration, Cell Proliferation. (2021) 54, no. 7, 10.1111/cpr.13057, e13057. 34028920 PMC8249791

[12] Medeiros M. , Candido M. F. , Valera E. T. , and Brassesco M. S. , The Multifaceted NF-kB: Are There Still Prospects of Its Inhibition for Clinical Intervention in Pediatric Central Nervous System Tumors?, Cellular and Molecular Life Sciences. (2021) 78, no. 17-18, 6161–6200, 10.1007/s00018-021-03906-7. 34333711 PMC11072991

[13] Xiao H. , Wang K. , Peng L. , and Yin Z. , Laquinimod Attenuates Oxidative Stress-Induced Mitochondrial Injury and Alleviates Intervertebral Disc Degeneration by Inhibiting the NF-κB Signaling Pathway, International Immunopharmacology. (2024) 131, 10.1016/j.intimp.2024.111804, 111804. 38457986

[14] Wang L. , Gu Y. , and Zhao H. , et al.Dioscin Attenuates Interleukin 1β (IL-1β)-Induced Catabolism and Apoptosis via Modulating the Toll-Like Receptor 4 (TLR4)/Nuclear Factor Kappa B (NF-κB) Signaling in Human Nucleus Pulposus Cells, Medical Science Monitor. (2020) 26, e923386–1-e923386-11, 10.12659/MSM.923386. 32841225 PMC7466834

[15] Chen Z.-B. , Yu Y.-B. , Wa Q.-B. , Zhou J.-W. , He M. , and Cen Y. , The Role of Quinazoline in Ameliorating Intervertebral Disc Degeneration by Inhibiting Oxidative Stress and Anti-Inflammation via NF-κB/MAPKs Signaling Pathway, European Review for Medical and Pharmacological Sciences. (2020) 24, no. 4, 2077–2086, 10.26355/eurrev_202002_20387. 32141577

[16] Chen H. , Shen G. , and Shang Q. , et al.Plastrum Testudinis Extract Suppresses Osteoclast Differentiation via the NF-κB Signaling Pathway and Ameliorates Senile Osteoporosis, Journal of Ethnopharmacology. (2021) 276, 10.1016/j.jep.2021.114195, 114195. 33974944

[17] Shen G.-Y. , Ren H. , and Huang J.-J. , et al.Plastrum Testudinis Extracts Promote BMSC Proliferation and Osteogenic Differentiation by Regulating Let-7f-5p and the TNFR2/PI3K/AKT Signaling Pathway, Cellular Physiology and Biochemistry. (2018) 47, no. 6, 2307–2318, 10.1159/000491541. 29975930

[18] Novais E. J. , Tran V. A. , and Johnston S. N. , et al.Long-Term Treatment With Senolytic Drugs Dasatinib and Quercetin Ameliorates Age-Dependent Intervertebral Disc Degeneration in Mice, Nature Communications. (2021) 12, no. 1, 10.1038/s41467-021-25453-2, 5213. PMC8417260 34480023

[19] Shang Q. , Yu X. , and Ren H. , et al.Effect of Plastrum Testudinis Extracts on the Proliferation and Osteogenic Differentiation of rBMSCs by Regulating p38 MAPK-Related Genes, Evidence-Based Complementary and Alternative Medicine. (2019) 2019, 10.1155/2019/6815620, 6815620. 30984279 PMC6431499

[20] Wang T.-T. , Chen W. , Zeng H.-P. , and Chen D.-F. , Chemical Components in Extracts From *Plastrum testudinis* With Proliferation-Promoting Effects on Rat Mesenchymal Stem Cells, Chemical Biology & Drug Design. (2012) 79, no. 6, 1049–1055, 10.1111/j.1747-0285.2012.01361.x. 22339978

[21] Xin M. , Ping Y. , and Zhang Y. , et al.Metabolomic and Lipidomic Profiling of Traditional Chinese Medicine Testudinis Carapax et Plastrum and Its Substitutes, Frontiers in Pharmacology. (2025) 16, 10.3389/fphar.2025.1549834, 1549834. 40206067 PMC11980632

[22] He Q. , Yang J. , and Zhang G. , et al.Sanhuang Jiangtang Tablet Protects Type 2 Diabetes Osteoporosis via AKT-GSK3β-NFATc1 Signaling Pathway by Integrating Bioinformatics Analysis and Experimental Validation, Journal of Ethnopharmacology. (2021) 273, 10.1016/j.jep.2021.113946, 113946. 33647426

[23] Reagan-Shaw S. , Nihal M. , and Ahmad N. , Dose Translation From Animal to Human Studies Revisited, The FASEB Journal. (2008) 22, no. 3, 659–661, 10.1096/fj.07-9574LSF. 17942826

[24] Nair A. B. and Jacob S. , A Simple Practice Guide for Dose Conversion Between Animals and Human, Journal of Basic and Clinical Pharmacy. (2016) 7, no. 2, 27–31, 10.4103/0976-0105.177703. 27057123 PMC4804402

[25] Tsingas M. , Ottone O. K. , and Haseeb A. , et al.Sox9 Deletion Causes Severe Intervertebral Disc Degeneration Characterized by Apoptosis, Matrix Remodeling, and Compartment-Specific Transcriptomic Changes, Matrix Biology. (2020) 94, 110–133, 10.1016/j.matbio.2020.09.003. 33027692 PMC7778523

[26] Liu Z. , Guo F. , and Wang Y. , et al.BATMAN-TCM: A Bioinformatics Analysis Tool for Molecular mechANism of Traditional Chinese Medicine, Scientific Reports. (2016) 6, 10.1038/srep21146, 21146. 26879404 PMC4754750

[27] Stelzer G. , Rosen N. , and Plaschkes I. , et al.The GeneCards Suite: From Gene Data Mining to Disease Genome Sequence Analyses, Current Protocols in Bioinformatics. (2016) 54, no. 1, 1–30, 10.1002/cpbi.5. 27322403

[28] Wu O. , Jin Y. , and Zhang Z. , et al.KMT2A Regulates the Autophagy-GATA4 Axis Through METTL3-Mediated m6A Modification of ATG4a to Promote NPCs Senescence and IVDD Progression, Bone Research. (2024) 12, no. 1, 1–18, 10.1038/s41413-024-00373-1. 39572532 PMC11582572

[29] Saul D. , Kosinsky R. L. , and Atkinson E. J. , et al.A New Gene Set Identifies Senescent Cells and Predicts Senescence-Associated Pathways Across Tissues, Nature Communications. (2022) 13, no. 1, 10.1038/s41467-022-32552-1, 4827. PMC9381717 35974106

[30] Wang T. , Huang S. , and He C. , Senescent Cells: A Therapeutic Target for Osteoporosis, Cell Proliferation. (2022) 55, no. 12, 10.1111/cpr.13323, e13323. 35986568 PMC9715365

[31] Che H. , Li J. , and Li Y. , et al.p16 Deficiency Attenuates Intervertebral Disc Degeneration by Adjusting Oxidative Stress and Nucleus Pulposus Cell Cycle, eLife. (2020) 9, 10.7554/eLife.52570, e52570. 32125276 PMC7065909

[32] Song C. , Zhou Y. , and Cheng K. , et al.Cellular Senescence – Molecular Mechanisms of Intervertebral Disc Degeneration From an Immune Perspective, Biomedicine & Pharmacotherapy. (2023) 162, 10.1016/j.biopha.2023.114711, 114711. 37084562

[33] Song C. , Hu P. , Peng R. , Li F. , Fang Z. , and Xu Y. , Bioenergetic Dysfunction in the Pathogenesis of Intervertebral Disc Degeneration, Pharmacological Research. (2024) 202, 10.1016/j.phrs.2024.107119, 107119. 38417775

[34] Ling Z. , Zeng X. , Luo Q. , Li X. , and Cui L. , Co-Morbid Mechanisms of Intervertebral Disc Degeneration and Osteoporosis: Biomechanical Coupling and Molecular Pathways Synergistically Driving Degenerative Lesions, Journal of Orthopaedic Surgery and Research. (2025) 20, no. 1, 10.1186/s13018-025-06075-6, 652. 40660249 PMC12261636

[35] Wang Y. , Cheng H. , Wang T. , Zhang K. , Zhang Y. , and Kang X. , Oxidative Stress in Intervertebral Disc Degeneration: Molecular Mechanisms, Pathogenesis and Treatment, Cell Proliferation. (2023) 56, no. 9, 10.1111/cpr.13448, e13448. 36915968 PMC10472537

[36] Yang Y. , Li H. , Zuo J. , and Lei F. , Mechanistic Interactions Driving Nucleus Pulposus Cell Senescence in Intervertebral Disc Degeneration: A Multi-Axial Perspective of Mechanical, Immune, and Metabolic Pathways, JOR SPINE. (2025) 8, no. 3, 10.1002/jsp2.70089, e70089. 40606198 PMC12216508

[37] Liang W. , Zhang S. , and Cai X. , et al.Inhibition of the JAK2/STAT3 Pathway Attenuates D-Galactose-Induced Nucleus Pulposus Cell Senescence and Intervertebral Disc Degeneration, Stem Cells International. (2025) 2025, 10.1155/sci/3373211, 3373211. 41497807 PMC12767374

[38] Xie C. , Shi Y. , and Chen Z.-H. , et al.Apigenin Alleviates Intervertebral Disc Degeneration *via* Restoring Autophagy Flux in Nucleus Pulposus Cells, Frontiers in Cell and Developmental Biology. (2022) 9, 10.3389/fcell.2021.787278, 787278. 35096819 PMC8795835

[39] Dong Y. , Li C. , and Tu S. , et al.Phosphatidylethanolamine Protects Nucleus Pulposus Cells From Oxidative Stress-Induced Cellular Senescence and Extracellular Matrix Degradation by Promoting Autophagy, JOR SPINE. (2025) 8, no. 2, 10.1002/jsp2.70058, e70058. 40309337 PMC12043014

[40] Su S. , Wu X. , and Li B. , et al.Inhibition of ERK1/2 Mediated Activation of Drp1 Alleviates Intervertebral Disc Degeneration via Suppressing Pyroptosis and Apoptosis in Nucleus Pulposus Cells, Journal of Orthopaedic Translation. (2025) 51, 163–175, 10.1016/j.jot.2025.01.013. 40160807 PMC11952795

[41] Lan T. , Shiyu-Hu , Shen Z. , Yan B. , and Chen J. , New Insights Into the Interplay Between miRNAs and Autophagy in the Aging of Intervertebral Discs, Ageing Research Reviews. (2021) 65, 10.1016/j.arr.2020.101227, 101227. 33238206

[42] Liu Y. , Dou Y. , Sun X. , and Yang Q. , Mechanisms and Therapeutic Strategies for Senescence-Associated Secretory Phenotype in the Intervertebral Disc Degeneration Microenvironment, Journal of Orthopaedic Translation. (2024) 45, 56–65, 10.1016/j.jot.2024.02.003. 38495743 PMC10943956

[43] Liao Z. , Su D. , and Liu H. , et al.Dihydroartemisinin Attenuated Intervertebral Disc Degeneration via Inhibiting PI3K/AKT and NF-κB Signaling Pathways, Oxidative Medicine and Cellular Longevity. (2022) 2022, 10.1155/2022/8672969, 8672969. 36120596 PMC9481359

[44] Luo L. , Gong J. , and Wang Z. , et al.Injectable Cartilage Matrix Hydrogel Loaded With Cartilage Endplate Stem Cells Engineered to Release Exosomes for Non-Invasive Treatment of Intervertebral Disc Degeneration, Bioactive Materials. (2022) 15, 29–43, 10.1016/j.bioactmat.2021.12.007. 35386360 PMC8940768

[45] Kang Y. , Li M. , and Hu B. , et al.Machine Learning Identifies Key Cells and Therapeutic Targets in Intervertebral Disc Degeneration: SASP-Driven Matrix Catabolism, Inflammation Amplification, and Metabolic Collapse, Inflammation. (2026) 49, 10.1007/s10753-025-02429-8, 34. 41501191 PMC12835096

[46] Zhou C. , Yao S. , and Fu F. , et al.Morroniside Attenuates Nucleus Pulposus Cell Senescence to Alleviate Intervertebral Disc Degeneration via Inhibiting ROS-Hippo-p53 Pathway, Frontiers in Pharmacology. (2022) 13, 10.3389/fphar.2022.942435, 942435. 36188539 PMC9524229

[47] Zhang P. , He J. , and Gan Y. , et al.Plastrum testudinis Ameliorates Oxidative Stress in Nucleus Pulposus Cells via Downregulating the TNF-α Signaling Pathway, Pharmaceuticals. (2023) 16, no. 10, 10.3390/ph16101482, 1482. 37895953 PMC10610230

[48] Ye S. , Zhong J. , and Huang J. , et al.Protective Effect of Plastrum Testudinis Extract on Dopaminergic Neurons in a Parkinson’s Disease Model Through DNMT1 Nuclear Translocation and SNCA’s Methylation, Biomedicine & Pharmacotherapy. (2021) 141, 10.1016/j.biopha.2021.111832, 111832. 34153844

[49] Lyu F.-J. , Cui H. , and Pan H. , et al.Painful Intervertebral Disc Degeneration and Inflammation: From Laboratory Evidence to Clinical Interventions, Bone Research. (2021) 9, no. 1, 1–14, 10.1038/s41413-020-00125-x. 33514693 PMC7846842

[50] Babkina I. I. , Sergeeva S. P. , and Gorbacheva L. R. , The Role of NF-κB in Neuroinflammation, Neurochemical Journal. (2021) 15, no. 2, 114–128, 10.1134/S1819712421020045.

